# Flow-Through Aptamer-SERS Microfluidic Platform for Aflatoxin B1 Detection in Food Crops

**DOI:** 10.1021/acsomega.6c05280

**Published:** 2026-06-26

**Authors:** Lorena Veliz, Betty Cristina Galarreta, François Lagugné-Labarthet

**Affiliations:** † Department of Chemistry, Western University, 1151 Richmond Street, London, Ontario N6A 5B7, Canada; ‡ Department of Sciences—Chemistry Section, Pontificia Universidad Catolica del Peru PUCP, Av. Universitaria 1801, Lima 32, Peru

## Abstract

Mycotoxins are secondary metabolites produced by fungal species. Due to its high toxicity, Aflatoxin B1 (AFB1) is one of the most hazardous mycotoxins found in various food crops. The ability to detect such a toxin is therefore important for the safe consumption and handling of large quantities of crops. For this, surface-enhanced spectroscopy provides the sensitivity to detect low concentrations of analytes but is limited in terms of specificity toward a given analyte. This work explores how to enhance the selectivity of a SERS active platform by conjugating it with aptamers designed to specifically recognize AFB1 target molecules. A robust flow-through lab-on-a-chip platform with an integrated SERS gold nanohole array functionalized with an aptamer (apta-SERS) was developed and tested on real samples, demonstrating rapid, high-affinity toxin detection. The sensitivity of the nanoholes was evaluated in the range of 10–700 ppb, and in the presence of a complex matrix such as food crop extracts, a limit of detection of 10 ppb was reached. Such a platform enables *in situ* AFB1 detection through SERS-enhanced aptamer recognition that forms a hairpin loop secondary structure around AFB1. The developed microfluidic platform minimizes direct contact with contaminated samples, and results are highly selective and reproducible, making it promising for other portable toxin-detection applications.

## Graphical abstract

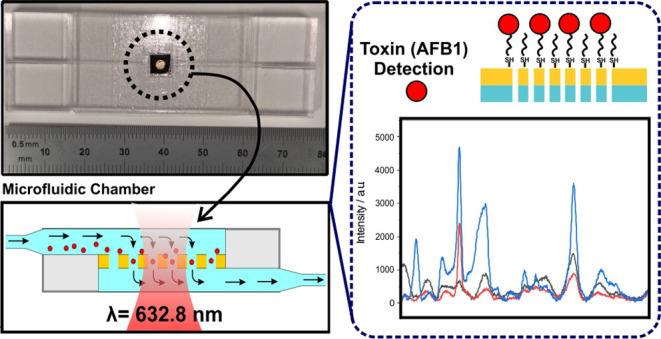

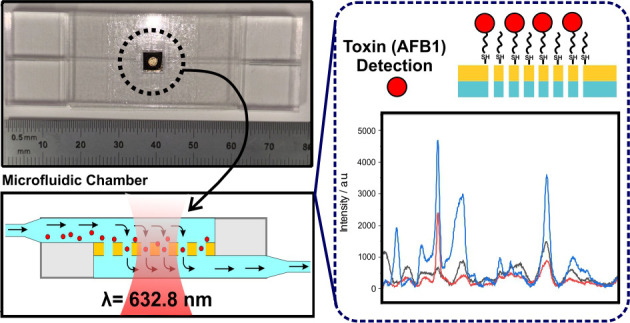

## Introduction

Aflatoxin B1 (AFB1) is one of the most frequent mycotoxins produced by certain fungi from the Aspergillus group and poses a significant threat to diverse agricultural commodities.^1^ AFB1 can affect maize, almonds, peanuts, rice, and even milk products, producing a variety of toxicological hazards.^2–5^ Recognized for its nephrotoxic, hepatotoxic, and carcinogenic effects,^6,7^ AFB1 has led to strict regulation throughout the world^8^ and, thus, the need to have innovative, reliable screening methods, which are essential to prevent contaminated batches from entering food and feed supply chains. The FDA limit for contaminated food is about 20 ppb, including corn/maize, rice, and others.^9^ Currently, different methods and techniques are used for AFB1 detection, such as high-performance liquid chromatography (HPLC) and gas chromatography (GC) combined with mass spectrometry (MS), fluorescence or ultraviolet detectors.^4,5^ However, despite providing high sensitivity and selectivity, these techniques require extensive sample preparation, skilled operators, and high-cost instrumentation, limiting their use in routine or on-farm testing. On the other hand, immunoassays such as ELISA are considered a good alternative because of their availability and a fast and simple detection system.^10^ Nonetheless, ELISA assays rely on antibodies with limited thermal and pH stability, compromising accuracy in variable agricultural environments.^11,12^ These challenges highlight the need for rapid, safe, cost-effective, and robust detection devices capable of deployment outside laboratories.

Optical sensing strategies provide attractive advantages for agricultural monitoring, including nondestructive analysis, portability, and low reagent consumption, together with acceptable limits of detection (LOD) and quantification (LOQ) that remain dependent on the technique and methodology used.^13^ Such optical analysis would potentially enable a first screening and quantification of aflatoxin at the sites before their gathering, thus preventing cross-contamination from the different batches. In this context, surface-enhanced Raman spectroscopy (SERS) offers the sensitivity needed for trace detection, particularly when spectra are analyzed in conjunction with numerical methods such as principal component analysis (PCA) and partial least-squares (PLS).^14^ However, the intrinsic lack of molecular specificity of SERS limits its broader applicability for real-world mycotoxin monitoring in complex matrices despite large local Raman enhancement.^15–19^

Several SERS substrates have been developed recently for the specific detection of AFB1 by synthesizing metallic nanoparticles (Au and Ag) through chemical reduction and electrochemical deposition.^20–23^ However, these bottom-up approaches may lack reproducibility, be susceptible to nanoparticle aggregation, and be difficult to integrate into controlled miniaturized fluidics systems. In this context, metallic platforms with nanohole arrays (NHA) have demonstrated superior performances, providing a homogeneous and reproducible surface that concentrates surface plasmons within the small individual nanoholes that can be exploited for enhanced optical measurements.^24^ At the same time, due to their geometry, NHA can act as traps, increasing the chances of collecting the target particles within the nanoscale holes.^17,25^ Through the years, it has been demonstrated that the sensor response is mostly dominated by the particles bound in the inner surface of the NHA rather than the particles attached to the top surface.^26^ This characteristic has highlighted the need to design systems that allow analytes to pass through the nanoholes and not just accumulate over the surface. In this regard, the development of microfabrication methods has enabled the design of novel flow-through sensing devices compatible with microfluidics chips. This flow-through model takes advantage of the design and trapping capabilities of NHA, transforming them into parallel microchannels that serve as a molecular gate to retain the desired target molecules.^27^ This characteristic of the flow-through configuration makes it unique compared with the traditional SERS platforms based on flow-over technologies and provides a suitable platform for the detection of low-concentration analytes such as mycotoxins. Several benefits of the flow-through architectures have been demonstrated in the past for a variety of fields, including proteins and cancer studies,^26,28^ highlighting a disruptive improvement in the response time, mass transportation, and diffusion limitations of nanofluids.^27,29^

Here, we developed a robust microfluidic, flow-through lab-on-a-chip device featuring a gold nanohole array selectively functionalized with a high-affinity thiolated DNA oligomer that recognizes AFB1 through a hairpin-loop secondary structure upon binding.^30^ These oligomers, also known as aptamers, offer several benefits over traditional bioreceptors, including chemical stability, reproducibility, ease of modification, and resistance to environmental changes such as temperature and pH, as well as superior sensitivity and selectivity.^13,31–33^ The flow-through configuration, combined with highly selective aptamers, enables real-time, in situ molecular recognition with enhanced spectral clarity.

PCA and PLS analyses of the collected spectra demonstrated that the apta‑SERS platforms maintain sensitivity across the AFB1 calibration range (10–700 ppb), achieving a limit of detection as low as 10 ppb. This performance was preserved even in complex food matrices such as maize, coffee, and rice extracts, underscoring the method’s suitability for real-world agricultural samples. The microfluidic integration of the flow-through SERS chip substantially reduces user exposure to contaminated materials while enabling rapid and automated measurements suitable for portable deployment. Concomitantly, the platform enables direct analysis of crude food extracts without extensive purification procedures, significantly shortening the overall workflow as compared to ELISA, HPLC, or LC-MS/MS. Also, in contrast to commercial lateral flow assays, the method does not rely on proprietary antibodies or enzymatic labels, reducing cost and simplifying operation.

Overall, this project establishes a foundation for a next-generation biosensing technology that unites molecular selectivity and plasmonic enhancement within a compact, field-ready analytical platform. Its modular design makes it a versatile device for expanding to additional high-risk contaminants, ultimately supporting safer food supply chains and more efficient monitoring practices.

## Experimental Section

### Production of Nanohole Arrays by Focus Ion Beam (FIB) Milling

Nanohole arrays (NHA) have been widely used as sensing platforms due to their relatively simple fabrication of nanoscale structures with specific plasmon modes.^34,35^ In this work, NHAs were used as the SERS-active substrate due to their well-defined plasmonic response and reproducible nanostructure geometry. Circular NHAs were fabricated by focused ion beam (FIB) milling on an Au-coated SiNx membrane and characterized by SEM (Figure 1a–c). FIB was chosen for its rapid and resist-free patterning process, which minimizes contamination and allows precise control over hole size and periodicity in comparison with bottom-up approaches. The resulting arrays consisted of circular holes (∼500 nm diameter) arranged over a (50 × 50) μm^2^ area, providing a uniform plasmonic surface suitable for SERS measurements. Full fabrication parameters are provided in the SI (Section SI-1).

**Figure 1:**
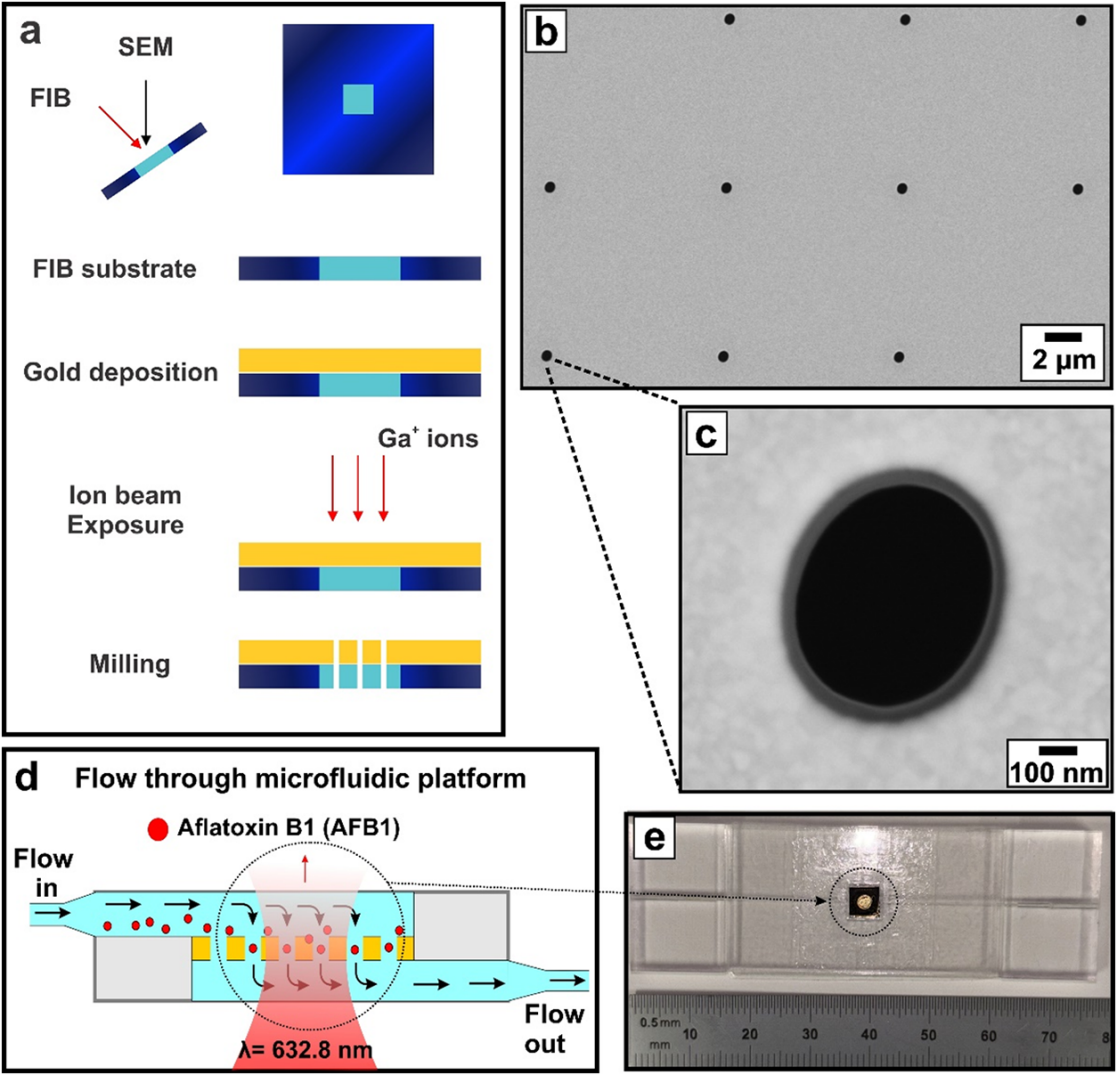
(a) FIB process scheme to fabricate the nanohole arrays in the SiNx substrate. (b) SEM image of the arrays in the gold substrate, and (c) zoom-in of a single 500 nm nanohole. (d) Microfluidic chamber scheme placed on the inverted optical microscope for Raman measurements. The flow in ensured by the combination of a syringe pump (flow in) and a peristaltic pump (flow out). (e) Photo of the microfluidic device with the SERS array at the center.

### Development of the Microfluidic Device

A 5 mm thick acrylic sheet with similar dimensions as the 3″ × 1″ microscope slide was used as a starting material, and the microfluidic channels were milled with a computer numerical controlled (CNC) milling machine. The apta-SERS chip (1 × 1 mm^2^) was centered on the slide within a 3 mm diameter chamber. The fluid channels were 250 mm in diameter and were inscribed by milling on both the top and the bottom face of the acrylic sheet. Holes were drilled at the two ends of the channels to accommodate the 250 μm syringe needles.

The SERS substrate was loaded into the microfluidic chamber and sealed using a seal film (Excel Scientific, Polyester 0.05 mm) and a glass coverslip on the optical side for the SERS measurements. Two needles (20 gauge diameter) and hoses were connected at each extremity of the chamber to allow the solutions to flow continuously in the microfluidic device during the experiments. The flow rate was adjusted with a syringe pump attached to the inlet and a peristaltic pump connected to the outlet during all the experiments. The microfluidic is compatible with an optical microscope (Figure 1d,e).

Overall, the use of FIB ensures consistent structural features across independently produced chips, and the incorporation of the flow-through NHA into a sealed microfluidic chamber allows a controlled sample introduction and detection under identical flow and exposure conditions for all measurements.

### Functionalization of the SERS Substrate with Aptamer

A thiolated-oligonucleotide 5′-GTTG​GGCA​CGTG​TTGTC​TCTC​TGTG​TCTC​GTGC​CCTT​CGCT​AGGC​CC-(CH_2_)_6_-SH-3′ previously reported by Guo et al.^36^ and selected through the SELEX method was purchased from Eurogentec and redissolved in ultrapure water for a final concentration of 100 μM. The thiol group at the extremity is crucial for binding the aptamer to the gold surface. This is the AFB1 recognition agent, also known as the AFB1 aptamer. A 10 μL aliquot of 10 μM AFB1-aptamer solution was then drop-casted on the substrate and allowed to dry overnight. The excess of solution was then eliminated by rinsing with ultrapure water. Finally, 10 μL of 1 mM of aqueous alpha-Methoxy-omega-mercapto hepta (ethylene glycol) (MeO-PEG(7)-SH Iris Biotech) solution was drop-casted right after passivating the surface for 2 h and then rinsed with ultrapure water. The freshly functionalized SERS substrate was inserted in the microfluidic chamber and loaded onto the Raman microscope.

### AFB1-Aptamer Assay by Raman Spectroscopy

AFB1 stock solution was prepared by dissolving 0.5 mg in 1 mL of methanol. Then, 93.6 μL of this solution was diluted in AFB1 buffer (10 mM Tris, 120 mM NaCl, 20 mM CaCl_2_ and 5 mM KCl, pH 7) for a final concentration of 10 μM. Different dilutions were prepared from the stock solution to test the performance of the AFB1-aptamer sensor in the calibration range of 10–700 ppb, with at least 3 replicates for each concentration. The solutions were flowed at 5 μL/min for 10 min using a syringe pump to facilitate contact between the toxin and the aptamer and encourage complex formation. Immediately afterward, the SERS spectra were collected over a (5 × 5) μm^2^ mapping area utilizing λ = 632.8 nm excitation, a 600 groove/mm grating, and a spectral range of 700–1800 cm^–1^. The Raman spectra of the functionalized samples with and without the toxin were collected with a laser intensity of 2 × 10^5^ W/cm^2^, 50 s acquisition time per spectrum and 1 accumulation. All experiments were carried out at ambient temperature to preserve the native conformation of the aptamer, as thermal fluctuations can disrupt secondary structure and reduce binding efficiency. The sensing assays were performed in a buffer adjusted to pH 7.0 to maintain aptamer stability and promote optimal AFB1–aptamer interaction. The use of neutral pH is consistent with conditions reported in foundational AFB1 aptamer characterization studies, such as Ma et al.,^30^ who examined AFB1 aptamer binding under physiological pH values (7.0–7.4). A fresh and new substrate was prepared for each concentration and replication to avoid residues and leftover species that could produce alterations in the measurements.

### AFB1 Assay in Real Samples (Coffee/Corn/Rice)

For all samples, 2 g of milled uncontaminated sample was first dried overnight at 100 °C to eliminate water residues. Then, in a 15 mL centrifuge tube, they were mixed with 10 mL of MeOH:H_2_O (70:30 v/v) and sonicated for 15 min at room temperature. After each sample was centrifuged at 4000 rpm for 1 h, the supernatant was collected. The extract was then filtered with a PTFE filter (0.42 μm) and kept at 4 °C until further use. Finally, samples were spiked by adding a known concentration of AFB1 standard solution. This protocol was commonly used as a standard procedure for ELISA assays to analyze mycotoxins in food.^37^

### Multivariate Analysis of AFB1 Samples: PCA and PLS

The SERS spectra (*n* = 3–8) for different concentrations of AFB1 obtained after the measurements were analyzed first by PCA using Orange software. Preliminary preprocessing of the SERS spectra included standardization and baseline correction. Then, 5 principal components were selected to explain 95% of the variance. On the other hand, the same group of SERS spectra was tested by partial least-squares (PLS) regression using SOLO v.9.5 (Eigenvector Research Inc.). SERS spectra were first preprocessed by using the same preprocessing applied for PCA. After that, all data were imported to SOLO and preprocessed using mean centering. Outliers were found using Q residuals and T2 Hotelling values. Cross-validation was done using the venetian blinds method. The variable selection was made automatically by the program based on variable importance in projection (VIP scores)/ signal-to-noise (s/n Ratio).

## Results and Discussion

### Preparation of the SERS Substrate (Au@Apt@PEG)

Unlike conventional static incubations or flow-over designs, the flow-through configuration was selected for the apta-SERS platform. As reported in previous studies,^24,27^ the principal advantage of the flow-through geometry is that the analyte directly moves accross the apta-SERS array, thereby increasing the likelihood and efficiency of molecular recognition within the microchannel. Several studies have demonstrated that this controlled microenvironment reduces diffusion limitations, improves binding kinetics, and may minimize the variability associated with manual sample handling.^24,26–28^ Nanoholes with a 500 nm diameter were chosen for their strong plasmonic resonance near the excitation wavelength, which leads to efficient electromagnetic field confinement and enhanced SERS activity. In addition, this dimension also facilitates the formation of reproducible hot spots and allows adequate interaction between the complex aptamer–AFB1 and the plasmonic surface. Finally, the gold nanoarrays were functionalized with the thiolated aptamer prior to their addition and sealing of the microfluidic chamber. The AFB1-aptamer was first prepared at a concentration of 10 μM, drop-cast onto the gold nanoarrays, and left to dry slowly overnight at room temperature to ensure that the strands would bind to the surface. The excess solution was eliminated by rinsing with ultrapure water to prevent nonspecific binding interactions. The surface was passivated with aqueous MeO-PEG (1 g/L) to fill the empty spaces between the holes and the surface, thereby enabling the localization of hotspots exclusively around the nanoholes without affecting the remainder of the substrate area. Figure 2a illustrates each step of the functionalization process, while Figure 2b displays the Raman spectra before and after surface functionalization. As anticipated, no signal was detected in the nanohole array inscribed in the gold substrate (black spectrum). Upon surface functionalization and rinsing with the AFB1-aptamer followed by MeO-PEG, the collected Raman spectra (blue spectrum) show clear evidence of surface functionalization. The observed spectral features can be attributed to the conformational dynamics of the aptamer upon target binding. In our study, the aptamer targeting AFB1 demonstrated a distinct hairpin loop^36^ configuration upon complex formation, consistent with previous reports. This structural rearrangement is critical, as aptamer-target interactions are often mediated by secondary structures such as hairpins, which facilitate selective binding. The vibrational changes detected through molecular spectroscopy, particularly in the phosphate backbone and sugar moieties, reflect these conformational shifts, underscoring the sensitivity of spectroscopic techniques not only to surface-bound functional groups but also to the three-dimensional folding of the aptamer upon analyte recognition.

**Figure 2:**
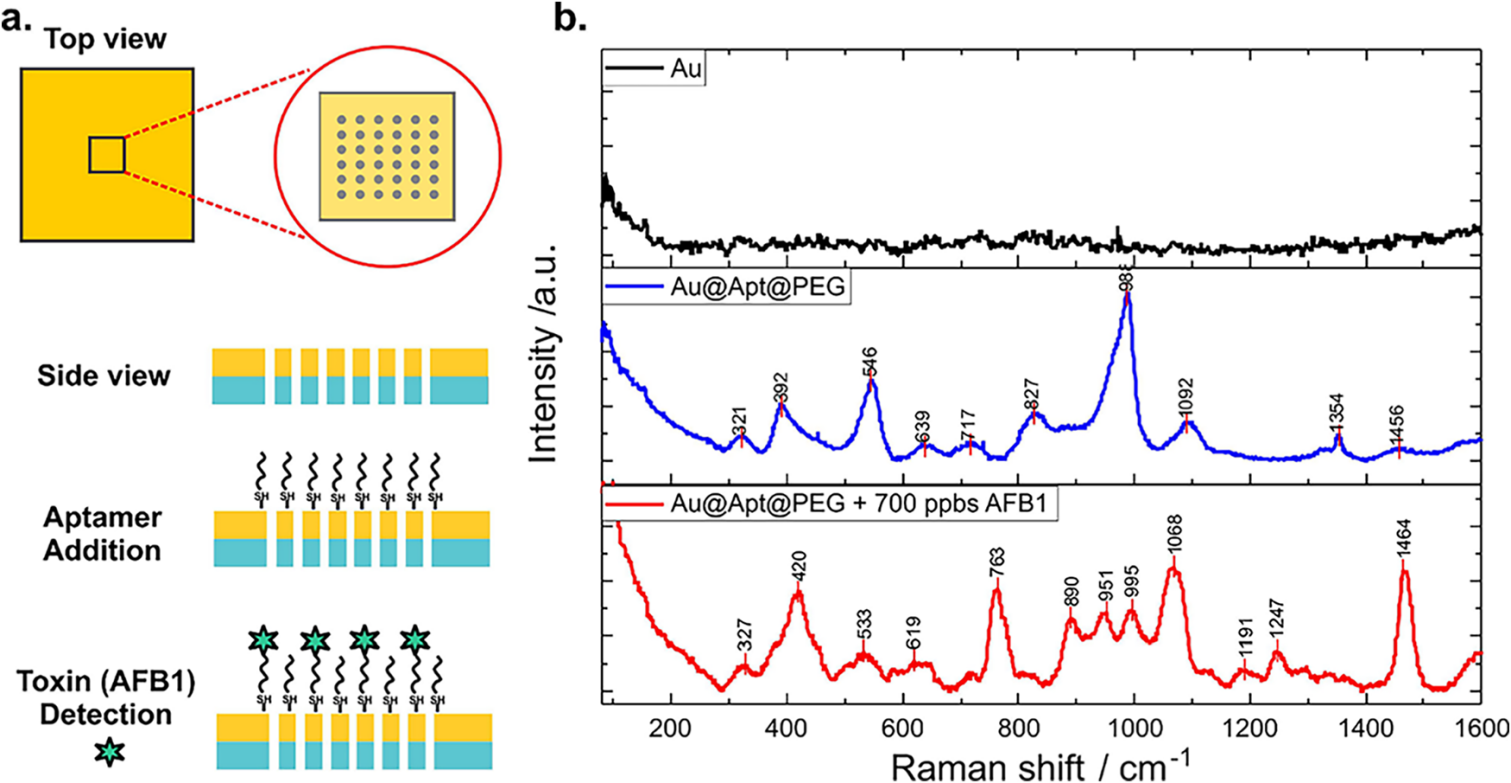
(a) Functionalization and detection process for AFB1 in the microfluidic chamber. (b) Raman spectra of bare nanohole array in the gold substrate (black), functionalized apta-SERS sensor (Au@Apt@PEG - blue) and after the addition of 700 ppb of AFB1.

The Raman spectrum for Au@Apt@PEG in Figure 2b shows several contributions, including a small shoulder at 321 cm^–1^, indicative of the interaction Au–S (the thiolate side of the aptamer).^15^ The signal at 639 cm^–1^ suggests that the vibrational mode of the ring breathing mode of guanine.^18^ A Raman stretching mode at 717 cm^–1^ is assigned to the ring breathing mode of adenine.^38^ Additionally, the signal around 827 cm^–1^ is linked to sugar vibrational modes.^39^ Interestingly, previous studies have reported an intense Raman band at 993–998 cm^–1^ due to the interaction and proximity of the aptamer to the gold surface.^40^ In this work, this band is observed at 988 cm^–1^ and can be attributed to the use of a slightly different aptamer compared to the reference, along with its unique method of adsorption on the gold surface, which may result in a change in the signal position. Following that, a vibration at 1092 cm^–1^ is due to the vibrational mode associated with the stretching of the PO^–^ backbone. Finally, the small signal at 1354 cm^–1^ can be attributed to the stretching in the adenine ring.^41^ The reproducibility of the flow-through SERS substrate after functionalization was confirmed by collecting *n* = 7 spectra around distinct nanohole arrays in different positions (Figure S1a). Additionally, the same experiments were conducted over several independent flow-through SERS substrates prepared on different days, highlighting a statistically relevant spectrum for Au@Apt@PEG (Figure S1b).

The introduction of a 700 ppb of AFB1 solution through the microfluidic (∼10 min) onto the apta-SERS sensor was conducted, highlighting a new spectrum (red spectrum in Figure 2b). The Au–S bond is still present but shifted to 327 cm^–1^, which allows us to conclude that the functionalization is stable even during the introduction of AFB1. New vibrational modes corresponded to a ring deformation in AFB1, and the Raman mode of the C–H bond (out of plane) was also observed at 619 and 763 cm^–1^. Concomitantly, the ring breathing ν­(C–O) is observed at 890 cm^–1^. Spectral difference between the functionalized nanoholes before and after the AFB1 solution was recorded in the region between 900–1100 cm^–1^. The mode at 988 cm^–1^ is replaced by two contributions at 951 cm^–1^, 995 cm^–1^, while the mode at 1092 cm^–1^ experiences a shift to 1068 cm^–1^. The 951 and 995 cm^–1^ bands are modes assigned to the bending and vibration in the C–O bond, while the 1068 cm^–1^ mode can still be attributed to the deoxyribose vibration in the aptamer complex. These spectral shifts are presumably due to the conformational change of the aptamer chain upon recognition of the target molecule. As it was reported in previous studies, this conformational change may cause a spectral shift to the new proximity of a TGT aptamer-fragment (in the active zone) to the surface once the mycotoxin is trapped.^40,42^ In our work, the conformational change can be explained by the shift from 1092 cm^–1^ to the more intense band at 1068 cm^–1^. Finally, a signal at approximately 1247 cm^–1^ corresponds to the out-of-plane bending vibration of the C–H ring, while the stretching vibration of the aromatic ring is observed at 1464 cm^–1^, both of which have been reported previously as AFB1 vibrational modes.^32^

To ensure the specificity of the aptamer/target interaction of the apta-SERS sensor, a SERS chip was functionalized with MeO-PEG, thus replacing aptamer chains and was tested with the same concentrations of the toxins. However, after adding the AFB1 with a concentration of 700 ppb, no signals were detected, indicating the effectiveness of the AFB1-aptamer complex in detecting the mycotoxin and the effectiveness of MeO-PEG to reduce the nonspecific interactions of the mycotoxin with the detection surface. (Figure S2).

In summary, thanks to the flow-through configuration, the apta-SERS system achieves more consistent spectral responses across replicates and operates with significantly lower sample volumes and shorter interaction times; these attributes are particularly important for rapid testing in agricultural protocols. At the same time, the controlled flow regime reduces hotspots associated with uneven drying or sample deposition, while the aptamer layer contributes a uniform chemical interface for analyte capture. These factors collectively may enhance measurement repeatability and address a key obstacle in translating SERS from laboratory demonstrations to applied monitoring tools.

### Detection of AFB1 by SERS and PCA Analysis

Despite the low concentration of AFB1 used in the previous section (700 ppb), the toxin’s signals remained detectable without the need for an external label. Lower concentrations of AFB1 (351, 79, 19, 10 ppb) were tested to determine the device’s limit of detection. Each standard was prepared by diluting the stock solution (10 μM) with an additional 50 μL of AFB1 buffer. The solutions were flowed for 10 min at 5 μL/min using a syringe pump to facilitate contact between the toxin and the aptamer and encourage complex formation. Figure 3a displays the average spectra for each concentration (*n* = at least 3 spectra per 6 concentrations in 3 different substrates), highlighting specific signals that decline along with decreasing mycotoxin levels.

**Figure 3:**
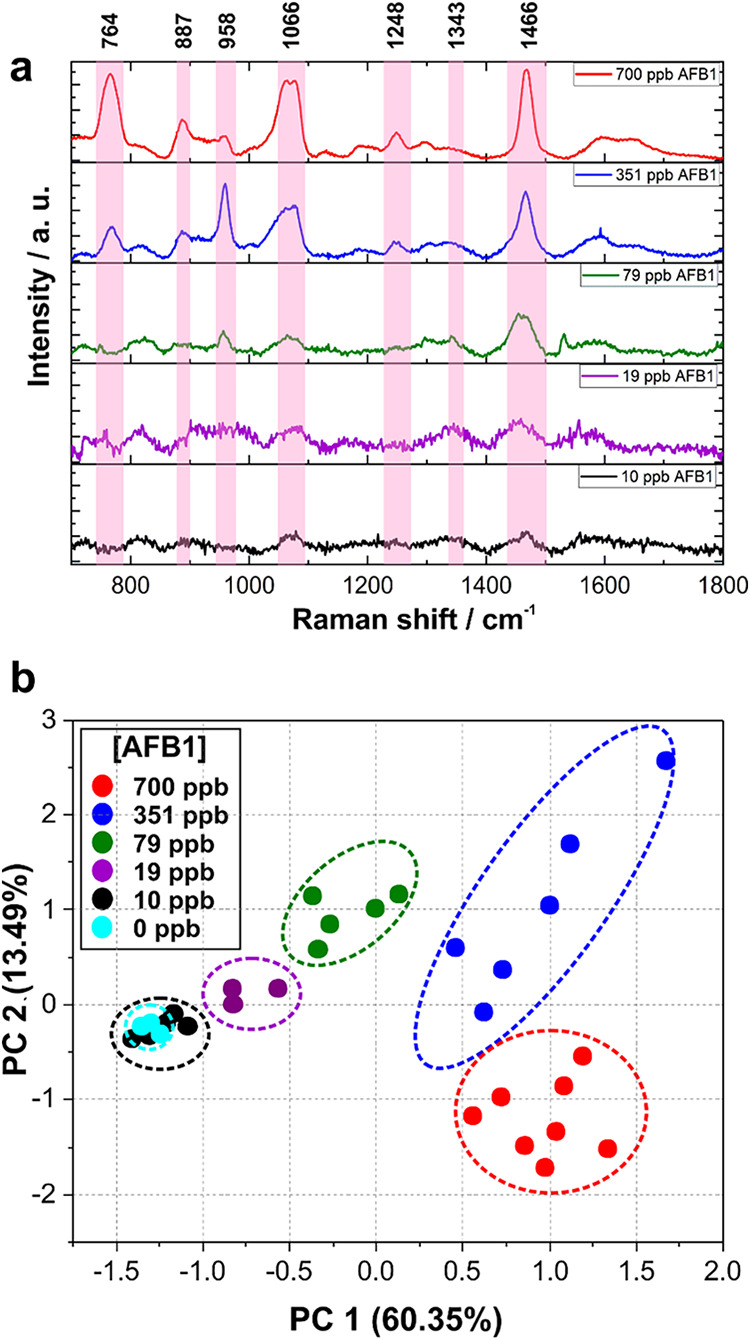
(a) Average Raman spectra of AFB1 across the tested concentration range. Each curve represents the mean spectrum of 3 spectra per concentration obtained from three different substrates. The pink-highlighted regions mark the diagnostic spectral intervals used in the PCA model, and the corresponding vibrational modes are annotated at the top of the plot. (b) PCA analysis for each concentration of AFB1 with PC1 and PC2 as the chosen components that allow the formation of the independent clusters.

The Au@Apt@PEG SERS sensor enables AFB1 detection through two complementary mechanisms that reflect how both the aptamer and the toxin interact with the plasmonic surface. When AFB1 binds to the surface-immobilized aptamer, the strand folds into a hairpin structure, altering its proximity to the metallic substrate and reducing the intensity of characteristic aptamer Raman bands at 887, 958, 1066, and 1343 cm^–1^.^39,40,42,43^ This decrease serves as an indirect indicator of toxin concentration, since fewer binding events occur at lower AFB1 levels, leaving the aptamer in a more extended, less SERS-active conformation. At the same time, AFB1 itself can generate direct SERS signals when molecules diffuse close to electromagnetic hotspots within the nanohole array, producing distinct peaks at 764, 1248, and 1464 cm^–1^.^19,32,44^ Because the likelihood of AFB1 occupying these hotspots diminishes as its concentration decreases, the intensity of these mycotoxin-specific bands also scales with molecular abundance. Together, the indirect aptamer response and the direct AFB1 signatures provide a dual-mode detection strategy that enhances sensitivity and strengthens analytical confidence.

As a result, the raw Raman spectra demonstrate that AFB1 detection is possible at 79 and 19 ppb; however, the spectra collected at lower concentrations (≤10 ppb) represent a challenge for conventional analysis. For this reason, PCA is of interest to further analyze the data set through a reduction of the data dimensionality, which can be exploited to reveal the aptamer-toxin complex into similar clusters. In this regard, the SERS data set was uploaded to the Orange Data Mining software to perform the PCA analysis. Around 3–8 spectra per concentration (*N* = 31) obtained from clean and freshly prepared different substrates (3 per concentration) were preprocessed for baseline correction. Then, the SERS spectra were standardized using a normal distribution with a mean μ = 0 and a standard deviation of σ^2^ = 1 to ensure consistency and homogeneity for further analysis. It is worth remembering that the principal advantage of PCA is to maximize the variance to form clusters. Thus, the PCA analysis was able to determine the pair of components that best differentiated between concentrations and the regions that better contribute to the model (highlighted in Figure 3a). In this case, PC1 and PC2 were selected, accounting for 60.3% and 13.5% of the variance, respectively (Figure 3b). Due to the good separation of the analyzed groups, we can conclude that there is a strong effect of the concentration of mycotoxin on the Raman spectra.

Following the regions identified by the PCA analysis, we also present the calibration curve established using three selected Raman modes. These modes, located at 764, 887, and 1066 cm^–1^, were integrated over the [735–755] cm^–1^, [855–980] cm^–1^, and [1020–1100] cm^–1^ spectral windows, respectively, taking only the area under the curve (AUC) of the Raman modes without any background. The concentration range from 0 to 351 ppb shows a linear variation as depicted in Figure 4, highlighted by *R* ^2^ values of 0.9746, 0.9948, and 0.9723 in each of the selected regions. This indicates a good sensitivity and linearity of the nanosensor and confirms its suitability for mycotoxin quantification for a 0–350 ppb dynamic linear range of concentrations. Above 350 ppb, the variation of the Raman signal deviates from linearity and no longer follows the linear trend, as can be observed in the SI section (Figure S3). Although subtle, this behavior aligns with expected saturation effects in aptamer-based SERS systems, where both aptamer binding-site availability and SERS hot-spot occupancy become limiting at elevated analyte levels.^30^ The limit of detection (LOD) was calculated as three times the standard deviation of the zero-value divided by the slope of the linear fit, resulting in 10 ppb (Figure 4).

**Figure 4:**
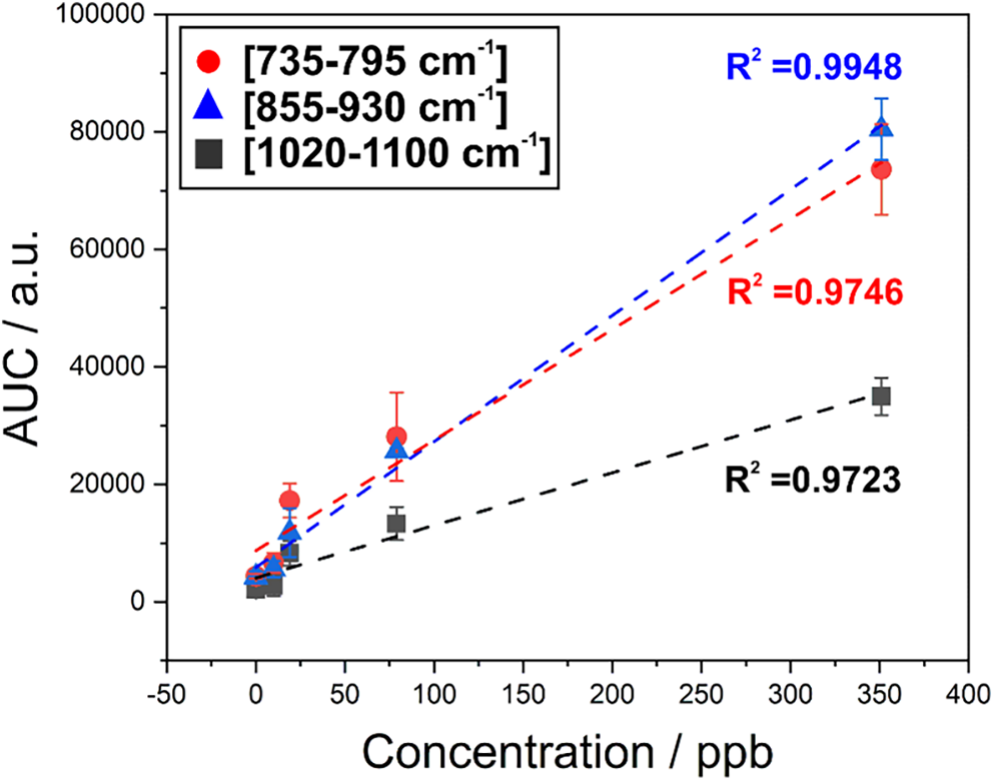
Calibration curve in the full range of AFB1 concentrations (0–351 ppb) with linear fitting in 3 different selected regions of the Raman spectra.

### Multivariate Analysis: Partial Least Square

As observed in the Raman spectra, the fingerprint obtained for AFB1 shows various vibrational modes resulting from the interaction of the toxin and the aptamer, as well as with the gold substrate. Additionally, the PCA analysis indicates that it is possible to differentiate between concentrations using machine learning techniques. To further evaluate the predictive performance and explore chemometric enhancement, Partial Least Squares Regression (PLSR) was applied in the calibration range of the data set (0–351 ppb). The model was evaluated using a 5-fold Venetian blinds cross-validation procedure. This approach ensured that each fold was representative of the total population (*N* = 31) with validation sets of 6–7 spectra. In this case, the same data set as in PCA was used, and the SERS spectra were preprocessed for baseline correction. Afterward, the data set was further preprocessed by applying mean centering to improve interpretation and mitigate bias, especially when high absolute values are present.

PLS allows the identification of the specific Raman bands that contribute to the model and are directly related to AFB1 concentration. The importance of these vibrational bands was determined automatically by the variable importance in projection (VIP scores), and it is shown in Figure 5a using a color gradient to highlight the most influential ones (in yellow). Here, two vibrational modes (764 and 1464 cm^–1^), coming from the AFB1, were identified with high VIP scores (around 3.5), as well as the signal at 1066 cm^–1^, which represents the change in conformation of the aptamer when the mycotoxin is trapped. Furthermore, two more signals related to the formation of the aptamer-AFB1 complex were obtained around a 1–1.5 VIP score, which also suggests the strong dependence of the model on the formation of the hairpin loop structure.

**Figure 5:**
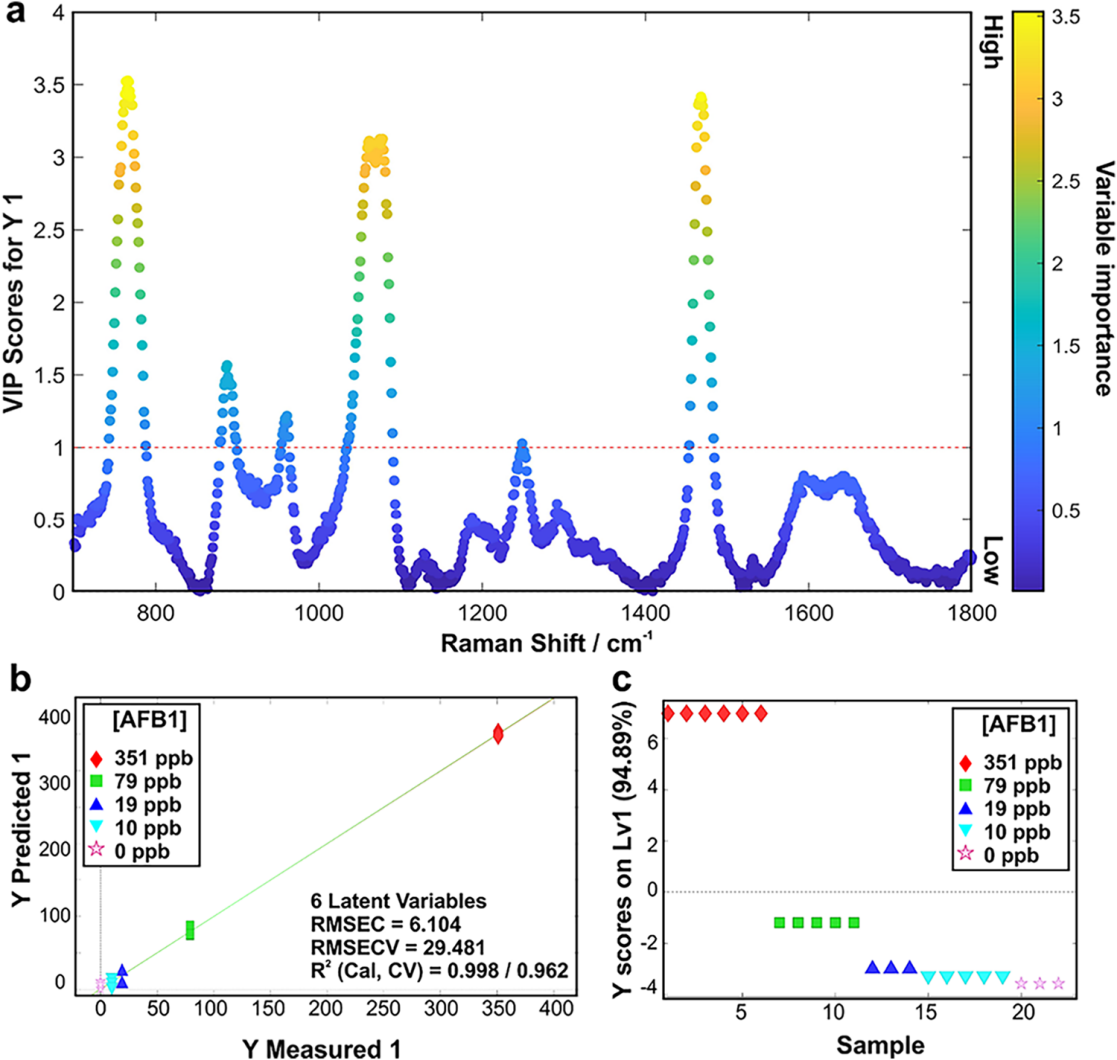
(a) VIP scores derived from the PLS model used for AFB1 quantification. The red dotted line marks VIP = 1, the standard threshold above which a variable (wavenumber) is considered to have a significant influence on the model. Raman shifts with VIP values above this line, shown in warmer colors (green to yellow), represent spectral regions that contributed most strongly to predicting AFB1 concentration. VIP scores were useful to calculate the (b) PLS regression curve for the apta-SERS sensor Au@Apt@PEG in the presence of different AFB1 concentrations and the (c) discrimination between concentrations given by the Y scores on LV1 plot resulting from the PLS model.

By using the regions with the highest VIP scores, a calibration curve (Figure 5b) was built using PLS regression with variable selection and 6 latent variables (LV) in the range of 700–1800 cm^–1^. Here, the linear regression achieved between the predicted and expected values was 0.9987 (*R*_cal_^2^), demonstrating the quality of the quantitative model. Moreover, strong calibration and cross-validation metrics were also obtained, with a root-mean-square error of calibration (RMSEC) of 6.10, a root-mean-square error of cross-validation (RMSECV) of 29.48, and an *R*_cv_^2^ of 0.960 (cross-validation), indicating high model accuracy and robustness. At the same time, Figure 5c shows the Y scores on LV1, which indicate a clear linear trend all over the sample. Scores on LV1 represent the main direction of variation in the data, and the samples with similar chemical profiles are clustered together, while the step-changes along LV1 axes reflect differences related to AFB1 concentration, validating the PLS model. In addition, PLS allows the identification of the specific Raman bands that contribute to the model and are directly related to AFB1 concentration.

Finally, the PLS model also calculated the difference between the Y-measured and the Y-predicted values (Y-residuals), which enables the quantification of the prediction error in the model, as well as evaluating its performance and determining the PLS _LOD_. Using the Y-residuals of the zero-value and the lowest concentration extracted from the PLS, the LOD was recalculated to evaluate the sensitivity of the model. In this regard, when derived from zero-value residuals, the resultant LOD was 10.9 ppb, closely matching the value obtained previously from the linear segment of the exponential curve. On the other hand, the Y-residuals from the lowest nonzero concentration (10 ppb) produced a lower LOD of 5.5 ppb, indicating that it is possible to improve sensitivity through multivariate analysis.

A potential limitation of the PCA and PLS analysis is the moderate number of replicate spectra per concentration (3–8, with a minimum of three to allow variance estimation). A larger data set would improve the robustness of these models. However, in the specific context of this study, the current replicate range proved sufficient to achieve the main analytical goals (discrimination of AFB1 concentrations and semiquantitative prediction). Within-substrate reproducibility tests (*n* = 3 at 79 ppb) yielded RSDs below 5% for AFB1-characteristic bands, indicating that few replicates capture the true spectral mean. Furthermore, PLS cross-validation produced a root-mean-square error of calibration (RMSEC) of 6.10, with a root-mean-square error of cross-validation (RMSECV) of 29.48, and an *R*_cv_^2^ of 0.96 (cross-validation), indicating high model accuracy and robustness. Readers should nonetheless interpret precise quantitative predictions (e.g., exact ppb values for unknown samples) with appropriate caution, and future studies are encouraged to adopt larger replicate sets (*n* ≥ 10) and multiple substrates per concentration to improve the robustness of the analyses.

The comparison between conventional curve fitting and chemometric techniques highlights the importance of multivariate analysis to refine detection limits. In our work, both approaches were applied to spectroscopic data to evaluate their performance in trace-level quantification of AFB1. In this regard, the PLS model commanded by the variable importance in projection (VIP) scores achieved a LOD between 5.5 and 10 ppb based on the Y-residual analysis. In contrast, the linear fitting model, constructed by using three spectral regions identified through principal component analysis (PCA), yielded an LOD of approximately 10 ppb. Thus, although PCA assistance for feature selection improved the relevance of input variables for linear modeling, the PLS approach demonstrated enhanced sensitivity and robustness, particularly in managing high-dimensional data. These results reveal the advantages of chemometric strategies like PLS in enhancing the analytical performance of sensor-based detection platforms for contaminants such as AFB1.

Overall, the flow-through apta-SERS nanosensor demonstrated strong analytical performance within the calibration range of 10–700 ppb AFB1. The LOD derived through chemometric modeling falls within or below several international regulatory thresholds for common commodities and aligns well with the requirements for practical *in situ* screening. As an example, the U.S Food and Drug Administration (FDA) reports an action level for AFB1 of 20 ppb in human food and animal feeds,^1,9^ making the flow-through apta-SERS nanosensor sufficiently sensitive for detecting aflatoxin concentrations below this regulatory limit. Compared with chromatographic methods, which provide excellent sensitivity but require extensive sample preparation and instrumentation, this flow-through platform offers a simplified procedure while maintaining meaningful quantitative capability. Its reproducible response across independent nanosensor batches further reinforces the robustness of the sensing approach and its potential utility in decentralized environments. In addition, we tested independent nanosensor chips that showed consistent Raman enhancement and repeatable spectral responses across the tested concentration range, demonstrating the reliability of the fabrication method.

### Analysis of Real Samples

One of the critical aspects of mycotoxin detection is that they usually originate in food crops, thus jeopardizing the integrity of the crops for safe consumption. The complex matrix of organic materials represents a challenge for Raman detection due to the numerous organic molecules present in the extracts, which can potentially attach to the aptamer and thus interfere with the detection of AFB1.^45^ Here, the high affinity of the AFB1-aptamer complex is of particular advantage because of its specificity, offering a simple and reliable methodology for the rapid detection of AFB1. This approach was tested against spiked natural extracts of rice, coffee, and maize that were selected as real samples (Figure 6a) because they are the most common food crops affected by AFB1 contamination. As proof of concept, extracts were prepared and filtered with PTFE. Then a known concentration of AFB1 was added to spike each extract to a final concentration of 79 and 351 ppb, and compared to AFB1-free samples. This step was crucial to avoid the loss of toxin during any step of the extraction process or filtration. Similar data pretreatment and variables for PLS analysis were used as described in the previous sections. Once again, the Y scores on the LV1 plot demonstrate that an excellent discrimination between the contaminated and uncontaminated real samples and standards is possible, validating this label-free apta-SERS approach. The clustering pattern observed in Figure 6b provides strong evidence for the selectivity of the proposed method when applied to real food matrices. Regardless of food crop type (rice, maize, or coffee), samples with the same AFB1 concentration clustered tightly together, while samples from different concentration levels remained well separated. This tendency indicates that the PLS model responds primarily to spectral variations in the concentration of AFB1 rather than to matrix-specific differences. This means that, despite the complex and different Raman backgrounds of rice, coffee, and maize, these matrix effects did not affect the output of the PLS model.

**Figure 6:**
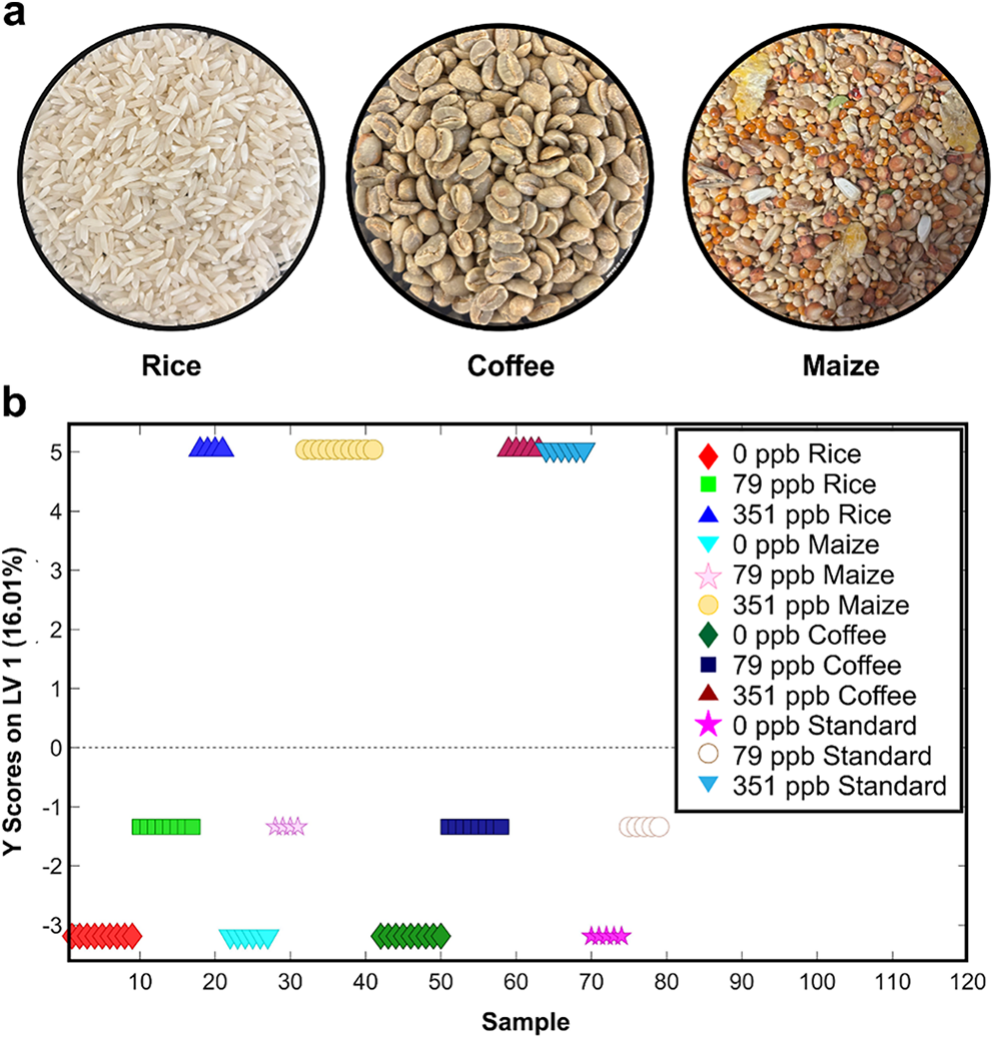
(a) Real samples for testing AFB1 in complex matrices. (b) Y scores on LV1 plot resulting from the PLS model in the absence and presence of 79 and 351 ppb of AFB1 for real samples and standards.

As shown in Figure 6b, all 0 ppb samples formed a consistent, low-score group, while samples containing 79 or 351 ppb AFB1 are shifted to positive score regions along LV1. This shift confirms that the model can reliably distinguish uncontaminated samples from those containing regulated or high mycotoxin levels, even when the samples originate from chemically and structurally diverse food crop matrices. Thus, the clear clustering across matrices demonstrates the robustness of the model and its potential for screening real-world agricultural products for AFB1 contamination. In addition, Figure S4 shows the Raman average spectra for each extracted sample with and without the presence of AFB1.

Overall, we demonstrated that the performance of the flow-through apta-SERS microfluidic platform may directly impact improving aflatoxin detection within agricultural and food supply chains. Our rapid, label-free, and simple analysis reduces testing time (10 min) compared to ELISA and chromatographic techniques (Table 1), enabling early detection of contaminated batches before aggregation, transport, or processing. The confined microfluidic design requires minimal sample volumes, reduces operator exposure to mycotoxins, and supports user-friendly operation even for nonexpert personnel. In terms of practicality, scalability, and potential for miniaturization, the microfluidic chamber is fabricated by milling an inexpensive acrylic sheet, and the sensing substrate is prepared using FIB milling. It is worth noting that despite requiring specialized instrumentation, FIB offers high precision with minimal reactant consumption and provides highly reproducible substrates, avoiding variations due to human handling. Due to the SERS sensing area ((50 × 50) μm^2^), even small substrates may be fabricated without altering the plasmonic functionality. At the same time, minimal volumes of sample are required (1 mL), enabling the future integration of the flow-through apta-SERS in portable systems. Conversely, the reusability of the apta-SERS and the long-term shelf life of the functionalized surfaces were not evaluated in this study; however, the durability and reproducibility of a similar apta-SERS sensor have been demonstrated previously for a different mycotoxin with minimal changes even after 2 weeks.^39^ Thus, we consider these are important areas to evaluate for future analysis. Finally, as shown in the previous sections, when paired with powerful characterization techniques such as SERS and chemometrics, the method is suitable for screening applications using minimal sample quantities. Such capabilities could help reduce cross-contamination, support compliance with regulatory standards, and improve safety at critical control points.

**Table 1:** Comparative Performances of the Developed Apta-Sensor and Existing AFB1 Detection Methods

Detection method	Label-free (label used)	Real samples	Substrate	Time of analysis	LOD (ppb)	Potential of Miniaturization	Refs
SERS	No (4-MBA)	Corn flour and oil	Colloidal NP	NS	0.039	No	^46^
SERS aptasensor	No (4-MPBA)	Wheat	Colloidal NP	NS	0.03	No	^47^
SERS aptasensor	No (4- PAPT)	Rice, corn, wheat	Colloidal and magnetic NPs	NS	0.24 × 10^–3^	No	^22^
FL	Yes	Peanut oil	Magnetic NP	NS	0.035	No	^30^
AFB1 ELISA Kit (E008)	Yes	Peanuts	commercial kit	<1 h	1	No	^48^
HPLC	Yes	Peanuts, corn and rice	Aptamer affinity column	HPLC runtime	0.02	No	^49^
UHPLC- MS/MS	Yes	Pure standards	HPLC column	25 min	0.04	No	^31^
LFIA	Yes	Oat milk	Nluc fusion proteins	NS	4.09	Yes	^50^
FL	Yes	Maize flour	Molecular Imprinted Polymers	NS	20	No	^51^
SERS + PCA	Yes	Maize	PDMS@AAO + Nanocauliflowers	NS	1.8 ppb	No	^52^
SERS + PCA–PLS	Yes	Rice, maize and Coffee	Nanohole Arrays in a Microfluidic Chamber	10 min	5.5–10	Yes	This work

For comparison, Table 1 lists recent approaches and sensors developed for AFB1 detection. This overview highlights the key differences in detection principles, time analysis, sensitivity levels, potential for miniaturization or portability, and applicability to real food matrices, enabling a clearer understanding of the advantages and remaining challenges associated with each approach. Presenting these methods side-by-side not only emphasizes the progress achieved by the present work, particularly in terms of sensitivity, selectivity, and matrix tolerance, but also clarifies how our platform complements or surpasses existing techniques.

## Conclusion

In this work, a flow-through label-free microfluidic device for accessible detection of AFB1 by SERS was developed. The uniformly fabricated nanohole array SERS platform embedded in a microfluidic channel was combined with aptamer recognition and multivariate data analysis (PCA and PLS). The flow-through microfluidic configuration significantly enhanced aptamer–analyte interactions, enabling reproducible SERS responses across independently fabricated nanosensors. This innovative approach enabled reliable detection and sensitivity in the range of 10–700 ppb and across different substrate batches. Moreover, we compared the LOD obtained using a conventional linear calibration curve based on the PCA dominant markers (LOD = 10 ppb for AFB1), with the LOD obtained through the multivariate PLS model, using Y-residual, which achieved a lower and more robust LOD of 5.5–10 ppb. This demonstrates that exploiting spectral information through multivariate analysis can enhance sensitivity and reliability, highlighting the clear advantage of PLS-based detection over conventional approaches. In addition, the microfluidic design allows significant reductions in sample volume (1 mL) and detection time (10 min), minimizing analyst exposure to contaminated samples, addressing key practical challenges in food safety monitoring. Real samples evaluation proved that this approach successfully enabled the detection of 79 and 351 ppb of AFB1 in complex matrices such as rice, maize and coffee. In conclusion, this work represents an important step in the development of point-of-care analysis by SERS detection with microfluidic devices for AFB1 and other label-free SERS screening assays.

## Supplementary Material



## Notes

Additional experimental details are summarized in this document (PDF)
