# Supplementary material for: Flow-Through Aptamer-SERS Microfluidic Platform for Aflatoxin B1 Detection in Food Crops
Source: ACS Omega. 2026 Jun 26;11(27):40905–15. doi: 10.1021/acsomega.6c05280 (PMC13382754; doi:10.1021/acsomega.6c05280)
Supplement: Supplementary file 1 [file ao6c05280_si_001.pdf]

Flow-Through Aptamer-SERS Microfluidic Platform for Aflatoxin B1 Detection in Food Crops

Lorena Veliz,[1] Betty Cristina Galarreta,[2,] François Lagugné-Labarthet[1,*]*

[1] Department of Chemistry, Western University, 1151 Richmond Street, London, ON, N6A 5B7, Canada.

[2] Department of Sciences - Chemistry Section, Pontificia Universidad Catolica del Peru PUCP, Av. Universitaria 1801, Lima 32, Peru.

Supporting Information

Section S1: Production of Nanohole arrays by Focus Ion Beam (FIB) milling.

Nanohole arrays (NHA) have been widely used as sensing platforms due to their relatively simple fabrication of nanoscale structures with specific plasmon modes. The use of advanced fabrication techniques allows for outstanding control of the shape and size, as well as the reproducibility of the metallic SERS structures. In this work, round-shaped nanohole arrays were fabricated by focused ion beam (FIB) and characterized by SEM. FIB was selected over electron beam lithography due to its clean and less time-consuming procedures. Furthermore, FIB does not require the use of a photoresist to create the pattern, so there is no chemical contamination due to resist residues at the end of the process. At the same time, FIB does not need extensive pre-treatments or substrate preparation in particular for gold, thus drastically reducing the fabrication time. The NHA were produced with a LEO 1540XB FIB/SEM microscope equipped with a Ga^+ ion beam to inscribe the holes over a gold thin film. Silicon nitride membranes (SiNx) with 100 nm thickness and 0.5 mm × 0.5 mm window size were selected as a substrate and covered with an 80 nm gold layer by electron beam deposition to make it suitable for SERS purposes. Gold was preferred for its stability compared to other common metals such as silver and copper. FIB milling was achieved with a beam energy of 30 keV and a current of 50 pA. The typical NHA were composed of circular holes of 500 nm in diameter over a surface area of (50 × 50) μm^2. Finally, the SERS substrate was stored until further use.

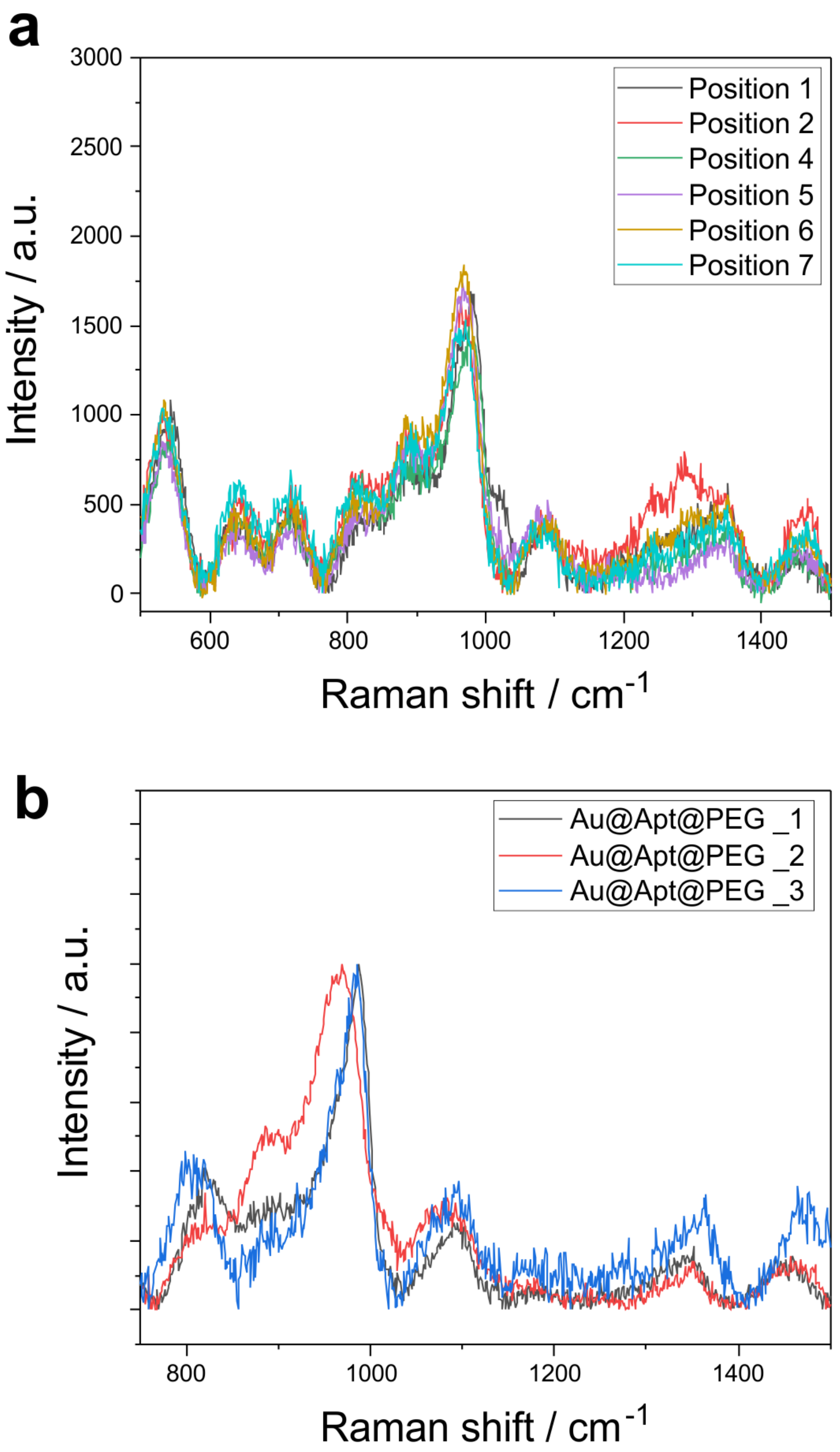

Figure S1. a) Raman spectra obtained in different positions on the same substrate. **b)** Raman spectra of different substrates after the functionalization process.

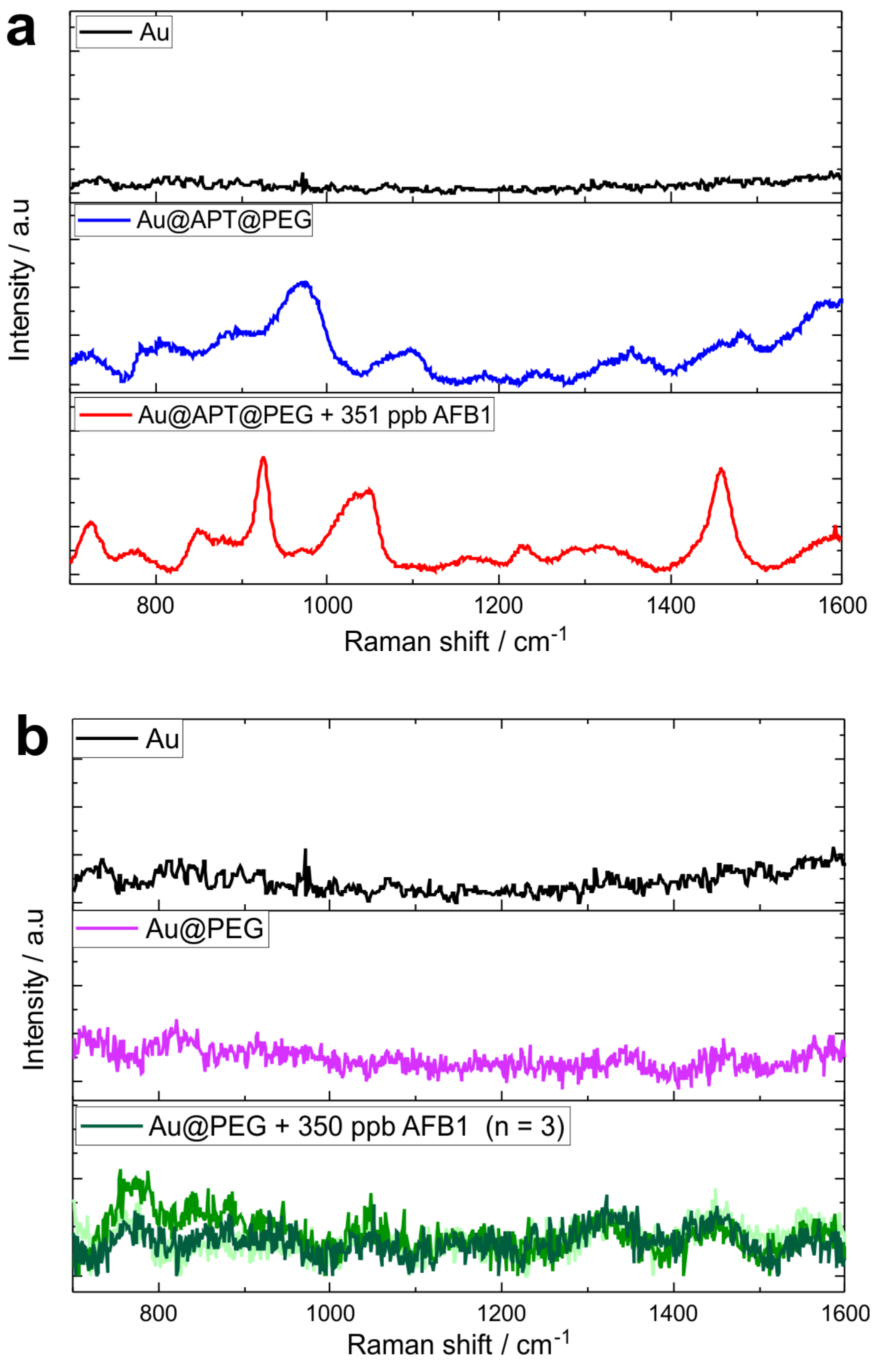

Figure S2. Raman spectra obtained for AFB1 detection when the Au substrate is functionalized with **a)** the aptamer and **b)** just the passivation agent (PEG)

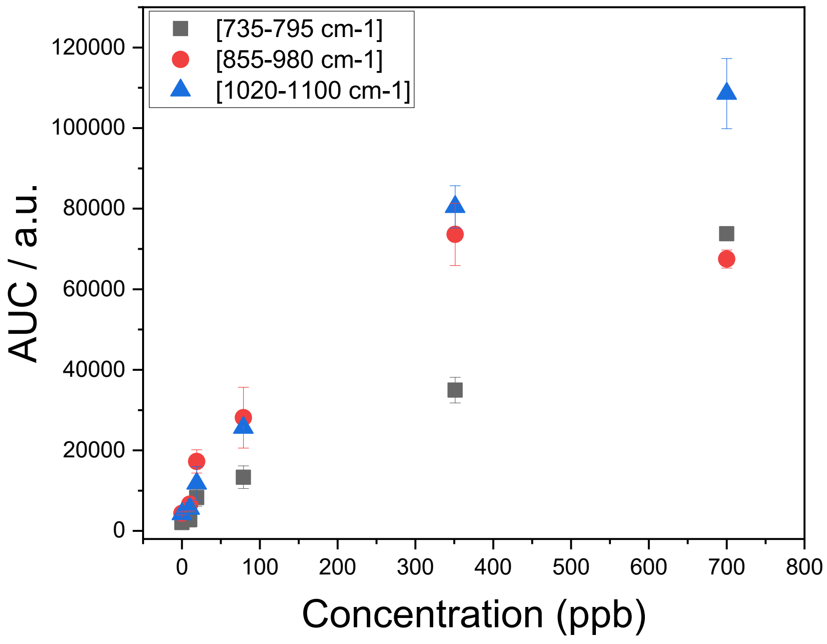

Figure S3. Calibration curve in the full calibration range (0-700 ppb) for three different peaks. Losing linear tendency is observed above 350 ppb to 700 ppb.

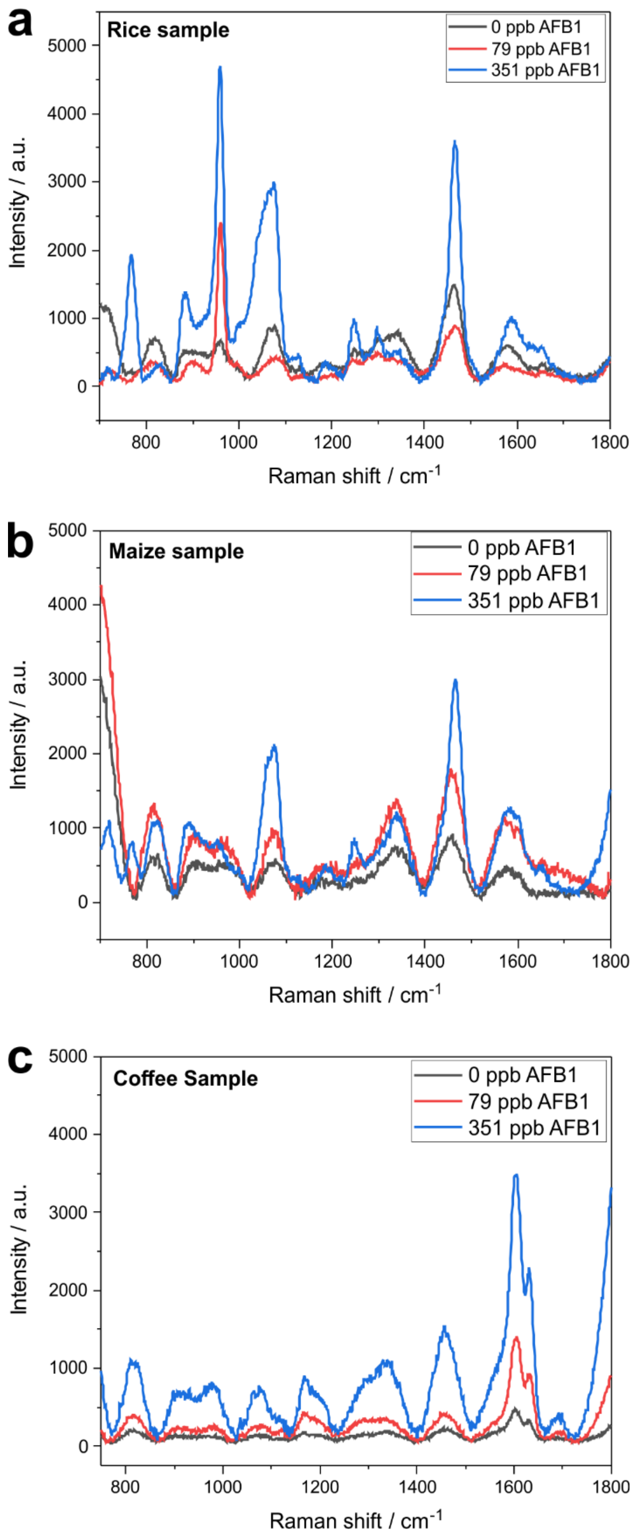

Figure S4. Raman average spectra (n ~ 5) for each real sample: **a)** commercial rice, **b)** commercial maize for birds and **c)** no-roasted Peruvian coffee beans.
